# Telephone-based screening tools for mild cognitive impairment and dementia in aging studies: a review of validated instruments

**DOI:** 10.3389/fnagi.2014.00016

**Published:** 2014-02-25

**Authors:** Teresa C. Castanho, Liliana Amorim, Joseph Zihl, Joana A. Palha, Nuno Sousa, Nadine C. Santos

**Affiliations:** ^1^School of Health Sciences, Life and Health Sciences Research Institute, University of MinhoBraga, Portugal; ^2^PT Government Associate Laboratory, ICVS/3B'sBraga/Guimarães, Portugal; ^3^Department of Psychology-Neuropsychology, University of MunichMunich, Germany

**Keywords:** neurocognitive impairment, early detection, rapid-assessment tools, dementia, telephone-based screening, cognition

## Abstract

The decline of cognitive function in old age is a great challenge for modern society. The simultaneous increase in dementia and other neurodegenerative diseases justifies a growing need for accurate and valid cognitive assessment instruments. Although in-person testing is considered the most effective and preferred administration mode of assessment, it can pose not only a research difficulty in reaching large and diverse population samples, but it may also limit the assessment and follow-up of individuals with either physical or health limitations or reduced motivation. Therefore, telephone-based cognitive screening instruments can be an alternative and attractive strategy to in-person assessments. In order to give a current view of the state of the art of telephone-based tools for cognitive assessment in aging, this review highlights some of the existing instruments with particular focus on data validation, cognitive domains assessed, administration time and instrument limitations and advantages. From the review of the literature, performed using the databases EBSCO, Science Direct and PubMed, it was possible to verify that while telephone-based tools are useful in research and clinical practice, providing a promising approach, the methodologies still need refinement in the validation steps, including comparison with either single instruments or neurocognitive test batteries, to improve specificity and sensitivity to validly detect subtle changes in cognition that may precede cognitive impairment.

## Overview

In the past years, improvements and progress in the health sciences have contributed to people living longer lives. In order to optimize physical and mental health, as well as well-being, during aging, appropriate gerontological research addressing changes in cognition is needed. Of note, aging is usually associated with an overall gradual decline in cognitive functioning, particularly in information processing/attention, memory and executive function, which may lead to a decrease in independence of daily living and, thus, of life quality (Salthouse, 2010). However, decline in cognitive domains is not uniform across all individuals, and even in the same individual, throughout aging (Riddle, 2007). For instance, factors that can possibly confer a risk of decline in cognitive performance, other than age in itself, are low(er) level of schooling, institutionalization, female gender, depressive mood, and the presence of “unhealthy” lifestyle factors and/or of clinical pathologies (Ardila et al., 2000; Van Gool et al., 2003, 2007; Wilson et al., 2009; Yamamoto et al., 2009; Paulo et al., 2011; Köhler et al., 2012; Santos et al., 2012; Costa et al., 2013; Viscogliosi et al., 2013). This heterogeneity in cognitive aging, and the need to reach larger population samples, challenges the available instruments that currently exist to efficiently assess global cognition and screen/detect deviations from healthy (“normal”) cognitive aging to cognitive impairments and dementia.

Cognitive impairment is defined as a clinical and transitional condition that spans from age-related memory impairment (AMI) to dementia (Petersen, 2004). Specifically, the American Academy of Neurology includes as criteria for mild cognitive impairment (MCI) the presence of memory complaints (preferably corroborated by an informant) and memory impairment, albeit still presenting normal global cognitive functioning and intact activities of daily life (ADL) (Petersen et al., 2001). Because cognitive impairments diagnosed as MCI are not severe enough to have a significant impact on daily life, individuals with MCI may be easily missed. Therefore, these individuals, who present a cognitive impairment, but without functional deficits, are at higher risk for dementia. Any possible intervention strategies to prevent this transition or, more precisely, the assessment of intervention strategies, requires the availability of valid and reliable screening assessment tools. At present, the gold standard to assess cognitive functioning, and to diagnose MCI and dementia, in older adults, is in-person (face-to-face) evaluation using a battery of standardized and validated cognitive tests (Herr and Ankri, 2013). However, this procedure implies high effort and time for the administration of the comprehensive cognitive battery by specialized researchers/clinicians. In addition, it holds also the risk of sampling biases because studies tend to comprise healthier and more educated older subjects, and those without limitations of mobility (Pachana et al., 2006). Therefore, cognitive assessment performed by telephone may provide an efficient, practical and valuable alternative and/or complementary strategy to the traditional face-to-face test administration methodology (Kliegel et al., 2007).

In fact, since the introduction of the Telephone Interview for Cognitive Status (TICS), by Brandt et al. (1988), cognitive screening instruments administered by telephone have been shown to provide some key advantages. Foremost, they not only allow a “rapid-administration” protocol that can be utilized by health care professionals, and/or researchers, on a regular basis (albeit being designed in a manner that can also be applied in the course of “normal” face-to-face evaluations if needed). Complementarily, they also serve as a more cost-effective and rapid-screening cognitive tools in medium- to large-scale epidemiological studies (Rabin et al., 2007), providing a means to lower dropout rates in longitudinal studies and overcoming geographical limits (Beeri et al., 2003). Furthermore, their indirect application is more likely to be well accepted by older/elder subjects who may present impediments in their physical mobility or health status, and/or with reduced motivation and, therefore, allow both “cognitive triage” and follow-up assessments in population samples that are difficult to reach (Kliegel et al., 2007). Finally, the practicability and efficiency of use is mainly derived from the instruments' design. The more accessible telephone instruments follow the model of the most widely accepted in-person brief screening measure for dementia diagnosis, the Mini-Mental State Examination (MMSE) (Wolfson et al., 2009).

The goal of this review is to provide a compiled base of the available telephone-based instruments for neurocognitive screening. Information is provided on the instrument purpose, authors, validation sample, and gold standard. Furthermore, characteristics regarding administration time, number of items, maximum score, cut-off threshold for cognitive impairment, and sensitivity and specificity measures are also provided. It is also reported if the instrument has been applied in other cohorts and/or translated to languages other than the original. Finally, an overall critical analysis is provided, informing on strengths and weakness of the instruments.

## Literature review

**Figure 1:**
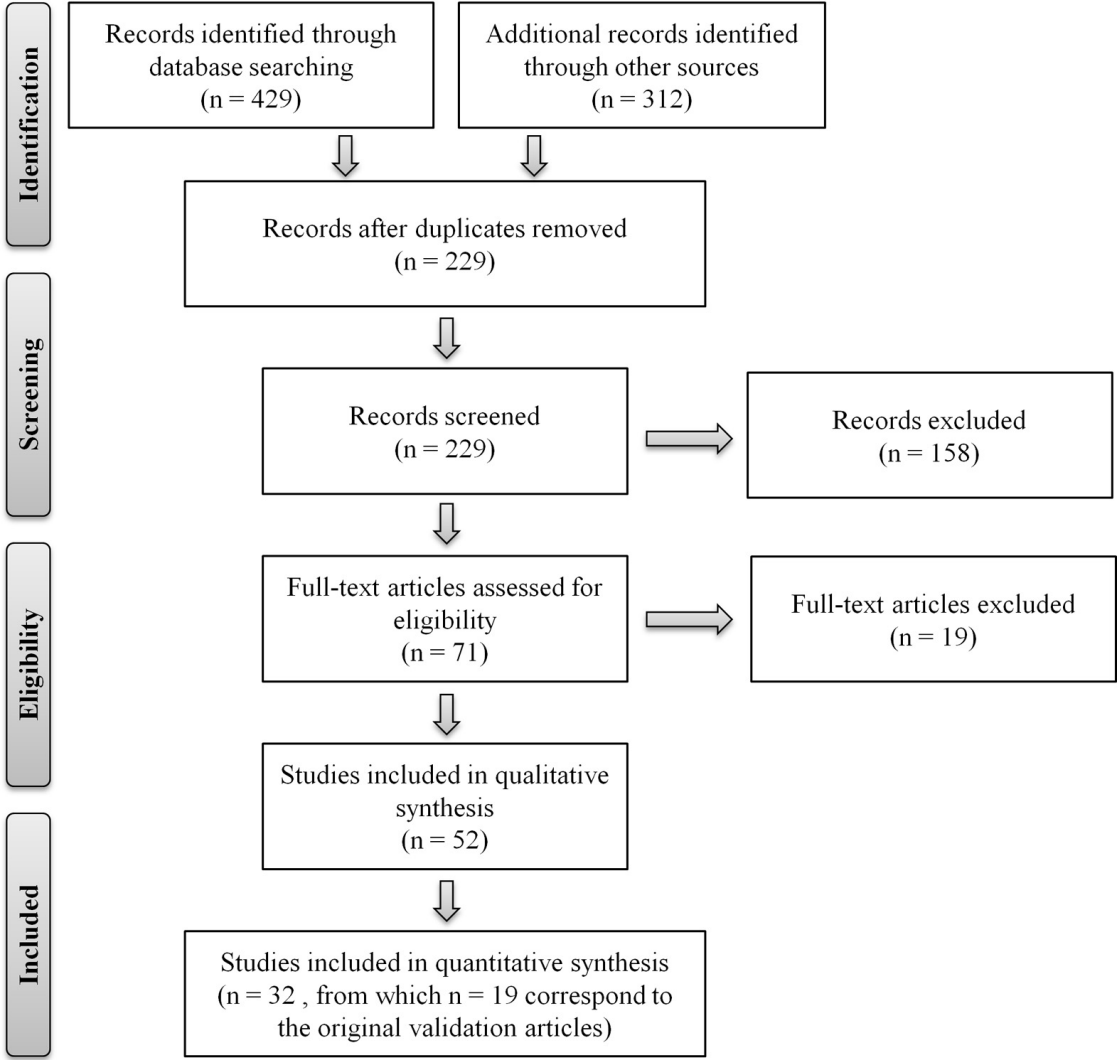
**Flow diagram of the literature review**. Depiction of the flow of information through the different phases of the review. PRISMA flow diagram template (Moher et al., 2009).

The systematic search of the literature was conducted in the EBSCO, Science Direct, and PubMed databases, using a combination of the search terms “telephone assessment,” “aging” (or “ageing”), “cognitive evaluation,” “MCI,” “dementia,” “telephone interview (for) cognitive status,” “validation questionnaire” and/or names of the specific instruments as these were identified. The search was limited to articles published in English and to instruments that allow discriminating between “normal” cognitive aging and cognitive impairment/dementia. Instruments/batteries administered by telephone to assess for cognitive function, and/or impairment, in non-aging cohorts, and/or designed for specific clinical cohorts [such as, for example, used to assess cognitive function in patients with pulmonary arterial hypertension (Taichman et al., 2005) or with chronic fatigue syndrome (McCue et al., 2002)], were not considered. Exceptions were for MCI, dementia, Alzheimer's disease, stroke and/or other cognitive-related disorders. Titles and abstracts were evaluated as a first step and then full-text articles were read for their relevance to this review. Two separate researchers conducted the search and the lists were cross-compared to generate a compiled list. The literature search was conducted between September 2012 and April 2013, with a total final of 19 separate telephone-administered screening tools identified. The search procedure is summarized in Figure 1, following the “Preferred Reporting Items for Systematic Reviews and Meta-Analyses (PRISMA)” flow diagram template (Moher et al., 2009). Table 1 summarizes the instruments, including number of elements/items and time of administration, and reporting on measures of validity. Furthermore, for each instrument, overall characteristics, usefulness and applicability, in clinical and research contexts, are next described.

**Table 1:** **Telephone-based neurocognitive screening instruments**.

**Original instrument**								
**Name and author**	**Validation sample**	**Country of validation**	**Statistical methods for analysis and validation**	**Gold standard^a^**	**Number of items; administration time**	**Cut-off for cognitive impairment/maximum possible score**	**Sensitivity; specificity**	**Validation in other countries**
Telephone Interview for Cognitive Status (TICS) (Brandt et al., 1988)	100 patients with mild DAT (mean age: 71 yrs), 33 healthy individuals (mean age: 67 yrs)	USA	Pearson correlation between MMSE an7 bhnd TICS scores; test-retest reliability; intraclass correlation coefficient	MMSE	11 items; 10 min	28/41	94%; 100%	Finland: 30 Alzheimer's disease patients, 26 healthy individuals (Jarvenpaa et al., 2002); Italy: 45 Alzheimer's disease patients, 64 healthy individuals (Dal Forno et al., 2006); Japan: 49 Alzheimer's disease patients, 86 healthy individuals (Konagaya et al., 2007); Spain: 36 stroke patients, 36 healthy individuals (Gude et al., 1994); Holland: 51 individuals (Kempen et al., 2007); Germany: 63 individuals (Loerbroks et al., 2008)
Hopkins Verbal Learning Test (HVLT) (Brandt, 1991)	129 healthy individuals (65 women and 64 men; aged 19–77 yrs)	USA	Cochran's *C* to calculate variances of the total recall scores; Pearson test to test correlations. Statistical tests of significance involving the amnesic group were not performed because of small sample size	Clinical assessment of dementia	12 items; 10 min	16/36	83%; 83%	France: 180 individuals (Rieu et al., 2006); China: 631 individuals (Shi et al., 2012)
Telephone version of the MMSE (ALFI-MMSE) (Roccaforte et al., 1992)	100 outpatients in a geriatric evaluation program (76 women and 24 men; aged ≥65 yrs)	USA	Pearson's correlation coefficients to compare the total scores of ALFI-MMSE and MMSE; McNemar's χ2 test to measure bias between the individual items and the two test versions; paired *t*-test to compare scores from participants with and without perceived hearing impairment and for the 22-item scores of the two test versions	MMSE and Brief Neuropsychiatric Screening Test	22 items; NR	17/22	68%; 100%	Brazil: 37 Alzheimer's disease patients, 36 healthy individuals (Kochhann et al., 2009); Italy: 104 Alzheimer's disease patients (Metitieri et al., 2001)
Telephone Assessed Mental State (TAMS) (Lanska et al., 1993)	30 individuals with DSM-III-R criteria for dementia (22 women and 8 men; aged 59–88 yrs)	USA	Spearman rank correlation and linear regression to assess the relation between TAMS and other instruments scores	MMSE	4 items; NR	4/17	NR; NR	NF
Modified Telephone Interview for Cognitive Status (TICSM) (Welsh et al., 1993)	209 individuals (aged 67–94 yrs^b^)	UK	Kruskal–Wallis statistic to assess differences between groups (normal, presumed normal, questionable and demented); Receiver Operating Characteristic (ROC) analysis to determine TICSM performance	MMSE	13 items; 5–10 min	27–30/39	99%; 86%	Israel: 576 individuals (Beeri et al., 2003); France: 120 women (Vercambre et al., 2010)
Short Portable Mental Status Questionnaire (SPMSQ-T) (Roccaforte et al., 1994)	100 individuals meeting DSM-III-R criteria for dementia (76 women and 24 men; aged ≥65 yrs)	USA	K statistic to evaluate the reliability of individual items; McNemar's χ 2 test to assess bias between the two routes of administration; Pearson's correlation to evaluate agreement between the two SPMSQ versions and the construct validity of the telephone SPMSQ in comparison to the face-to-face version and the MMSE; criterion validity measured by comparing sensitivity and specificity of SPMSQ versions to the clinical diagnosis of dementia	In-person evaluation	10 items; NR	NR/10	74%; 79%	NF
Blessed Telephone Information—memory—concentration test (TIMC) (Kawas et al., 1995)	84 individuals (31 men and 45 women; aged 50–98 yrs)	USA	Spearman's rank correlation coefficient to assess correlation between in-person and telephone assessment; paired *t*-tests to determine differences; multiple regression analysis to determine the covariates that could predict the model	Blessed Information Memory (IMC)	27 items; 5–10 min	NR/NR	NR; NR	NF
Telephone Screening Protocol (TELE) (Gatz et al., 1995)	30 outpatients, 26 individuals randomly selected (aged ≥55 yrs^b^)	USA	Standard receiver operating characteristic (ROC) analysis	Mental State Questionnaire (MSQ)	10 items; NR	15–16/20	86%; 90%	NF
Structured Telephone Interview for Dementia Assessment (STIDA) (Go et al., 1997)	15 individuals with cognitive impairment, 13 healthy individuals (22 women and 8 men; aged 60–88 yrs)	USA	Internal consistency measured using correlation between each STIDA subscale with the total STIDA score using the Informant/Subject STIDA (no information regarding the type of correlation test or its name was given by the authors); ROC curves contrasting the behavior of three tests, using clinician-based CDR as the gold standard, were generated	CDR rating scale for dementia	6 subscales; 10 min (if no medical information is collected)	NR/NR	93%; 77%	NF
Telephone Cognitive Assessment Battery (TCAB) (Debanne et al., 1997)	40 patients with DAT, 40 healthy individuals (48 women and 32 men; mean age 75 and 71 yrs, respectively)	USA	Shapiro Wilk's test to evaluate normality of data; *T*-tests to compare means of independent and paired samples; Kappa statistic to assess interrater reliability; discriminant analysis techniques to evaluate the TCAB's ability to classify individuals according to cognitive status	Expert opinion	6 neuropsychological tests; 15–20 min	NR/NR	97.5% (cases) and 92.5% (controls); 85.0% (cases) and 97.5% (controls)	NF
Memory Impairment Screen Telephone (MIS-T) (Buschke et al., 1999)	27 individuals with dementia, 273 healthy individuals (≥65 yrs^b^)	USA	Receiver operating characteristic (ROC) curves for each screening measure were generated to plot the advantage/disadvantage of sensitivity and specificity; for each of the screening measures, discriminative validity was assessed by calculating the sensitivity and specificity for detecting dementia for various test cut-cores given different base rates; area under the ROC curve (AUC) to compare screening tests; McNemar test to determine statistically significant differences in specificities between tests at constant values of sensitivity; ROC curves to evaluate the sensitivity-specificity for all dementias vs. no dementia	DSM-III-R	4 items; 4 min	4/8	78%; 93%	NF
Telephone adaptation of the Modified Mini—Mental State Exam (T3MS) (Norton et al., 1999)	263 community dwelling elderly (aged 65–93 yrs^b^)	USA	Repeated measures ANOVA to assess telephone and in-person administrations; regression techniques to develop a model of translation of T3MS to 3MS scores; Pearson correlation coefficients to assess 3MS—T3MS agreement with 10 cognitive domain categories and the agreement of the overall tests scores and 3 subscales	Modified MMSE version (3M)	34 items; NR	NR/100	91%; 97%	NF
Minnesota Cognitive Acuity Screen (MCAS) (Knopman et al., 2000)	99 mild to moderate dementia, 129 community-dwelling elderly individuals (aged 55–85 yrs^b^)	USA	Analysis of variance to determine overall group differences in demographic characteristics, in-office neuropsychological test performance, and performance on the MCAS subtests; Pearson product–moment correlations between MCAS total score and in-office neuropsychological measures; receiver operator characteristic (ROC) curves to evaluate overall classification accuracy; ordinal logistic regression with adjacent category logits to generate predicted probabilities for MCAS total scores using age and education as covariates and then regenerated covariate-specific ROC curves to identify whether sensitivity and/or specificity could be improved after controlling of these factors	NR	9 subtests; 15 min	NR/60	98%; 99%	NF
The 26-point telephone version of the Mini-Mental Status Examination (TMMSE) (Newkirk et al., 2004)	46 patients with DAT (24 women and 22 men; aged 55–90 yrs)	USA	Correlation coefficients computed (tests used not reported); linear regression with predictor variables centered and interaction terms included; 2-tailed paired *t*-test to test whether scores were higher on one version than the other; McNemar's chi-square test with the exact function to identify inconsistencies for individual items	MMSE	26 items; 5–10 min	NR/26	NR; NR	China: 34 Alzheimer's disease patients, 31 healthy individuals (Wong and Fong, 2009)
Brief Screen for Cognitive Impairment (BSCI) (Hill et al., 2005)	35 individuals with dementia; 35 healthy individuals (34 women and 36 men; aged 65–89 yrs)	USA	Comparisons of the differences between cases and controls in BSCI scores; comparisons of the correlations between patient scores on BSCI; comparisons of the areas under the receiver operating characteristic (ROC) curves	MMSE and Alzheimer's Disease Assessment Scale (ADAS)	3 items; 80 s	NR/NR	77%; 97%	NF
Brief Test of Adult Cognition by Telephone (BTACT) (Tun and Lanchman, 2006)	84 healthy community-dwelling individuals (aged 23–80 yrs^b^)	USA	Test scores were excluded for outliers that were >2.5 SD from the age-group mean or failure to follow instructions; Kolmogorov–Smirnov tests; ANOVA for testing differences; Tukey tests; to examine the effects of age after controlling for education effects, educational level included as a covariate	NR	6 subtests; 15–20 min	NR/NR	NR; NR	NF
Cognitive Telephone Screening Instrument (COGTEL) (Kliegel et al., 2007)	81 younger adults (40 women and 41 men, 19–37 yrs;) and 83 older individuals (41 women and 42 men, 59–75 yrs)	Germany	Significance level of 0.05; ANOVA to test variance effects of age and administration mode; effect sizes calculated; factor analyses to assess factorial structures; extraction of principal component factors using an eigenvalue of less than 1; factors orthogonally rotated with the Varimax procedure; confirmatory factor analysis; to evaluate concurrent validity correlations between COGTEL scores, age and education, and external cognitive indicators were computed; Kolmogorov–Smirnov test to determine distribution	Wechsler Memory Scale-Revised (WMS-R) and Wechsler Adult Intelligence Scale-Revised (WAIS-R)	6 subtests; 13–14 min	NR/NR	NR; NR	NF
Memory and Aging Telephone Screen (MATS) (Rabin et al., 2007)	120 older individuals with MCI and/or memory complaints (75 women and 45 men; aged ≥60 yrs)	USA	Skew and kurtosis statistics to determine asymmetry and peakedness in the distribution of MATS scores; parametric tests two-tailed tests, and nonparametric equivalents, utilized in analyses involving discrimination scores; ANOVA to evaluate group differences in MATS scores on the subjective memory test; *Post-hoc* comparisons using the Tukey HSD correction; One-Way analysis of covariance (ANCOVA) to evaluate mean differences; MANOVA to evaluate group differences in MATS scores; Pearson correlation coefficients	Consensus between neuropsychologists and a geropsychiatrist	12 items (subjective memory questionnaire) and 10 items (learning test); 20 min (subjective memory questionnaire and learning test combined)	30/50	NR; NR	NF
Telephone Montreal Cognitive Assessment (T-MoCA) and Short version of Telephone Montreal Cognitive Assessment (T-MoCA-Short) (Pendlebury et al., 2013)	91 patients with minor stroke or transient ischemic attack^b^	UK	Differences between MoCA face-to-face and T-MoCA evaluated through the Wilcoxon signed rank test. Area under the receiver—operating characteristic curve to predict mild cognitive impairment by T-MoCA	Montreal Cognitive Assessment (MoCA)	22 items (T-MoCA) and 12 items (T-MoCA-Short); NR (T-MoCA and T-MoCA-Short)	18–19/22 (T-MoCA) and 10–11/22 (T-MoCA-Short)	81% and 89% (cut-off 18 and 19, respectively, T-MoCA) and 70% and 96% (cut-off 10 and 11, respectively, T-MoCA-Short)^c^; 59% and 46% (cut-off 18 and 19, respectively, T-MoCA) and 51% and 39% (cut-off 10 and 11, respectively, T-MoCA-Short)^c^	NF

*CDR, Clinical Dementia Rating; DSM-III-R, Diagnostic and Statistical Manual of Mental Disorders, third edition, revised; DAT: Alzheimer's dementia; MMSE: Mini-Mental-State Examination; yrs: age in years; MCI: Mild Cognitive Impairment; NF, Not Found, unable to find any published peer-reviewed articles regarding instrument translations and/or validations in other countries; NR, Not Reported, information not provided by the authors*.

a *Gold Standard: empirical frame of reference against which an individual's test performance is compared*.

b *No information provided regarding gender and/or age*.

c *Sensitivity and specificity for the cut-off values; however, authors also provide sensitivity and specificity values for other scores*.

## Telephone-based neurocognitive screening instruments

### Telephone interview for cognitive status (TICS) and telephone interview for cognitive status—modified (TICSM)

The TICS instrument was originally purposed for the evaluation of cognitive functions in patients with Alzheimer's disease (Brandt et al., 1988; Brandt, 1991). Surpassing its original intent, it is now the most frequently used telephone-based cognitive screening test in medium-to large-scale studies and epidemiologic surveys (Herr and Ankri, 2013). Briefly, it assesses orientation to time and place, attention, short-term memory, sentence repetition, immediate recall, naming to verbal description, word opposites and praxis. The TICSM is the TICS modified version, adding a measure of delayed verbal recall. Both instruments have been translated into several languages, including Finnish, French, German, Hebrew, Italian, Japanese, and Spanish, serving an important role in clinical and research contexts. In clinical trials, TICS discriminated carefully diagnosed Alzheimer's disease patients from healthy controls, and in a sample of stroke patients TICS and TICSM were found valid instruments for evaluating cognitive function (Brandt et al., 1988; Barber and Stott, 2004). In epidemiological studies, both instruments detected a range of mild to moderate cognitive disorders and appeared to have comparable sensitivity and specificity as cognitive screening instruments for dementia and Alzheimer's disease (Welsh et al., 1993; Smith et al., 2008; Wolfson et al., 2009). There is, however, relatively little information concerning its use in longitudinal studies (Lopez and Kuller, 2010) and with older individuals (Baker et al., 2013). Both the TICS and TICSM' scores are highly correlated (*r* = 0.94, *p* < 0.001 and *r* = 0.57, *p* < 0.05, respectively), with the MMSE (Brandt et al., 1988; Jager et al., 2003). Furthermore, TICSM scores are adjusted to subjects' educational level (Gatz et al., 2002) and it is assumed to distinguish reliably between normal cognition, MCI and dementia (Knopman et al., 2010). Despite its wide use, the only information reported on its application is indicated in the TICSM validation study, in which psychometricians applied the instrument.

The original study indicates some key strengths of the instrument, including: cost-effectiveness for use in large-scale studies; little evidence of ceiling and/or practice effects; and greater acceptability by participants, who appear to find the telephone interview less threatening than if conducted in a face-to-face clinic assessment. These indicate for the possibility of lower dropout rates in trials and longitudinal studies. Another strength relates with the fact that it addresses the lower end of the cognitive ability spectrum (dementia) (Kliegel et al., 2007). Still, the authors also report on some important instrument limitations, namely: individuals with hearing impairment may be unable to complete the test or make errors; certain words are more difficult for participants to distinguish clearly and require careful attention to pronunciation; concentration and recall repetition may be hindered if words are not clearly heard. Finally, the authors also note that the instrument must be validated in preclinical populations, so to assess for the positive predictive value in healthy or mildly impaired or “ambiguous” cases.

### Telephone version of the mini-mental status examination (ALFI-MMSE) and the 26-point adaptation of the ALFI-MMSE (T-MMSE)

This telephone-based version of the MMSE was originally administered as part of the Adult Lifestyles and Function Interview (ALFI) (Roccaforte et al., 1992). It has been recommended as an instrument to examine cognitive function in patients with Alzheimer's disease, mainly regarding orientation, attention, memory recall, and calculation, and as means to reassess individuals who have been evaluated in person, allowing to assess cognitive stability or estimating decline. The ALFI correlated significantly with other face-to-face screening tests, specifically the MMSE (*r* = 0.85; *p* < 0.001) and the Brief Neuropsychiatric Test (correlation and *p*-values not reported) (Roccaforte et al., 1992). The ALFI-MMSE has been used in several studies including stroke patients (Longstreth et al., 2010), post-myocardial infarction patients (Thomas et al., 2011), and older adults with different levels of nutritional risk (Roberts et al., 2007), as a practical assessment tool when face-to-face assessments were difficult to obtain. Subsequently, a 26-point adaptation of the ALFI-MMSE was constructed (the T-MMSE), which in addition comprised a three-step action-based response (“say hello, tap the mouthpiece of the phone three times, then say I'm back”) and the request to provide the phone number (Newkirk et al., 2004). The T-MMSE was validated in a group of patients with probable Alzheimer's disease (Newkirk et al., 2004), although the authors mentioned that future research using a randomized test order would be necessary to better estimate the effect of previous exposure to the test (test–retest). For both tests, the ALFI-MMSE and the T-MMSE, a clinical nurse specialist conducted the phone interviews.

### Telephone assessed mental state (TAMS)

Designed by Lanska et al. (1993) and adapted from the MMSE, this brief instrument is a compilation of 4-items that assess for orientation to time and place, attention and memory. In the population sample of Alzheimer's disease patients, the TAMS' scores correlated well with those of instruments administered face-to-face; specifically, the TAMS was found to correlate significantly with the MMSE (*r* = 0.81, *p* < 0.001). Instrument scores were not independent of education. The authors report the instrument as inadequate for dementia diagnosis, or to assess subtle deficits, except in the context of a comprehensive clinical evaluation. The instrument is recommended to be administered by a trained psychometrician.

### Short portable mental status questionnaire (SPMSQ)

The SPMSQ is a 10-item test that measures the presence and severity of cognitive impairment regarding orientation to time and place, memory for personal information and serial subtraction (Roccaforte et al., 1994). It is a brief and easy instrument to administer that allows discriminating between subjects with dementia or without dementia. The SPMSQ telephone version was found to significantly correlate (*r* = 0.81, *p* < 0.001) with face-to-face MMSE (Smith et al., 2008). Regarding test application, no information was provided concerning specific qualifications and/or skills required.

### Blessed telephone information-memory-concentration test (TIMC)

The telephone version of the Blessed IMC consists of a 27-item list to evaluate verbal memory and orientation to time and place, which highly correlated with its face-to-face version (Spearman rank = 0.96, *p* < 0.001) (Kawas et al., 1995). The telephone-version has a greater acceptance by participants, compared with its face-to-face form, since the time needed for assessment is shorter. Despite being a potential instrument for epidemiological and longitudinal cognitive research, the authors recommend its administration in a broader and more varied population sample for further validation. No information is provided regarding specific qualifications recommended for test applicants and/or interviewers.

### Telephone screening protocol (TELE)

The TELE was introduced by Gatz et al. (1995) and it is based on the 10-item Mental Status Questionnaire (Kahn et al., 1960), with an additional 11 items concerning attention, verbal short-term memory and cognitive abstraction, and health issues. Clinical psychologists, registered nurses or other professionals with medical background carried out the screening in the validation process (Gatz et al., 2002). On sensitivity and specificity measures it discriminated reliably between Alzheimer's disease patients and healthy cognitively normal individuals (Gatz et al., 2002), particularly in the verbal working memory (digits backwards), 3-word recall, and indication of current month (Jarvenpaa et al., 2002). Initially designed to address the lower end of the cognitive ability spectrum, which is considered an advantage, it is consequently not as suitable in studies addressing normal cognitive aging (Kliegel et al., 2007), perhaps due to ceiling effects.

### Telephone cognitive assessment battery (TCAB)

The TCAB was designed to evaluate elderly cognitive status, which can discriminate between mild cognitively impaired and cognitively normal individuals (Debanne et al., 1997). It comprises six categories concerning mental status, semantic memory, reasoning, executive ability and language. This instrument requires administration by a well-trained professional, preferably from the field of neuropsychology. It is noted by the original authors that further work is needed to indicate for its applicability in wider community settings.

### Structured telephone interview for dementia assessment (STIDA)

Developed for the NIMH Genetic Initiative Alzheimer's Disease Study Group, and administered by skilled clinicians, the STIDA is a brief telephone screening instrument designed to discriminate people with normal cognitive functioning from those with cognitive changes due to early Alzheimer's disease (Go et al., 1997). It comprises items from the MMSE and from the Blessed-Orientation-Memory-Concentration (BOMC), and consists of six subscales including memory, orientation to time and place, judgment, and community and home activities. A much-abbreviated version of STIDA has been developed that contains questions on cognitive abilities and functional status (Go et al., 1997); however, no published studies regarding its application were found.

### Memory impairment screen telephone (MIS-T)

Based on the well-known in-person Memory Impairment Screen (MIS), this test contains the same semantic memory elements as the Free and Cued Selective Reminding (FCSRT) and the Double Memory Tests (DMT) (Lipton et al., 2003). The MIS-T was originally designed to measure episodic memory (Buschke et al., 1999) in a validation sample of randomly selected older individuals, and has been applied in cross-sectional studies. In the validation study individuals were evaluated by a neurologist, a neuropsychologist and a social worker student (Lipton et al., 2003). Used independently and in conjunction with the Category Fluency Test (semantic memory), and with other telephone cognitive screening tests, namely the TICS, the MIS-T demonstrated to be more robust than the TICS in discriminating dementia from normal cognitive functioning (Smith et al., 2008).

### Minnesota cognitive acuity screen (MCAS)

The MCAS comprises nine cognitive domains, including orientation to time and place, attention, delayed word recall, comprehension, sentences repetition, naming, computation, judgment, and verbal fluency (Knopman et al., 2000). The analysis of each domain score can be used to discriminate individuals with cognitive impairment due to MCI or Alzheimer's disease from cognitively normal subjects. The MCAS has also been proven an effective screening instrument for memory disorders (Tremont et al., 2011). Regarding the test administration, no information is provided regarding applicants' qualifications.

### Hopkins verbal learning test (HVLT)

The HVLT questionnaire assesses various aspects of verbal memory: short and long delay recall and recognition (Brandt, 1991). Six distinct forms of HVLT are available, each containing a 12-item unique word list, which can be used to avoid practice effects due to repeated administration. Although it was designed for face-to-face application, the HVLT can also be administered by telephone (Brandt, 1991). The reliability and validity of the instrument has been verified in patients with traumatic brain injuries, schizophrenia and in most common subtypes of dementia (Alzheimer's disease and vascular dementia) (Kuslansky et al., 2004). No information is provided concerning specific skills recommended for test applicants.

The HVLT has as main advantages its appropriateness for serial testing as part of longitudinal studies, with the possibility for alternative forms to be used to circumvent practice effects (test–retest). Also, the results were independent of the effects of demographic variables, and as such it may be more appropriate for identifying memory deficits associated with dementing processes than the MMSE. As shortcomings of the instrument, the authors refer to its validation in samples of convenience (patients referred to geriatric psychiatry and for neuropsychological evaluation); therefore, the authors indicate for the necessity of further research in other community settings. It is also noted that the HVLT performance is compromised in persons with low reading ability (perhaps, as a proxy of education), as well as in individuals diagnosed with clinical depression. Further research is also recommended in order to develop shorter, efficient versions, or in combination with other tools to improve the sensitivity of the instrument.

### Telephone adaptation of the modified mini-mental state exam (T3MS)

The T3MS is a modified version of the 3MS for the assessment of orientation to time and place, verbal memory, mental flexibility, abstract reasoning and language. The outcome of the T3MS correlates with the 3MS (*r* = 0.82; *p*-value not reported). The T3MS can distinguish between patients with MCI and individuals without cognitive impairment, with a sensitivity of 82% and a specificity of 100%, and was found a reasonable substitute for its face-to-face version (Alexopoulos et al., 2006). This instrument was used in the Cache County Study, designed to examine factors associated with the risk of Alzheimer's disease and other forms of dementia (Norton et al., 1999). No information is provided regarding qualifications of the interviewers, and no comments are made regarding how it compares with other instruments. The authors recognized that more studies are necessary to extrapolate the results to individuals with moderate to severe cognitive impairment, as well as studies with a more homogeneous population.

### Brief screen for cognitive impairment (BSCI)

The BSCI consists of a three-item test (delayed verbal recall, frequency of help with planning everyday activities, and frequency of help remembering to take medications), which is also suitable to detect dementia (Hill et al., 2005). The target sample of the original study comprised demented patients and cognitively healthy individuals. Compared with other screening tests, the BSCI has the advantage of being very brief and presenting no difficulties in its administration and scoring. However, the authors recommend that an experienced interviewer should conduct the evaluation. In addition, it should not to be used in a stand-alone evaluation of MCI or dementia (Fillit et al., 2003). The main advantage of the instrument is that it was designed to be included in initiatives targeted to the elderly population, including comprehensive disease management and geriatric case management programs. Specifically, as described by the original authors, the BSCI can be incorporated into larger telephonic health risk assessments (in the original study those conducted by Medicare-managed care plans). No information is available regarding its application in other studies and/or cohorts.

### Brief test of adult cognition by telephone (BTACT)

The BTACT is a brief test that covers several domains of adult cognition (Tun and Lanchman, 2006). Specifically, it assesses speed of processing, verbal working and long-term memory, executive function, and reasoning. Notably, the instrument can be paired with an optional computerized task-switching test, yielding further information on reaction time and executive function. The items are based on laboratory research and telephone versions of well-known psychometric testing instruments (these were not, however, specified). Furthermore, it was used as a part of the MIDUS-II (Mid-Life in the United States) study, which consisted of a sample of 7000 healthy individuals between 35 and 85 years of age (Tun and Lanchman, 2005). One important feature of this test is its applicability in well-functioning younger and middle-aged individuals as well as older people, from a range of educational backgrounds, and also in face-to-face evaluations, providing versatility in aging studies that span different age intervals (Tun and Lanchman, 2006). The authors recommend recording of the telephone interview and that, as hearing loss can compromise performance, a brief screening on hearing should be conducted at the beginning of the interview. No information is provided concerning specific characteristics and/or qualifications of the applicants and/or interviewers.

### Memory and aging telephone screen (MATS)

Developed and designed purposely by Rabin et al. (2007) for longitudinal assessment, the MATS consists of a subjective cognitive complaints questionnaire on subjects' perceived cognitive decline (onset, course, severity and impact on functioning), and 10 cognitive items assessing verbal memory (list-learning). The instrument was not modeled after the MMSE and excluded items known to lack sensitivity in preclinical groups (e.g., orientation to person or place, basic expressive language, praxis), being designed to screen individuals with MCI and/or significant cognitive complaints. It provides a key advantage over other instruments by including both objective and subjective memory assessments, and also by not showing a ceiling effect (even in cognitively intact controls). It is also reported that education, gender, and depressive symptoms did not significantly influence the results. Still, it is noted by the authors the relatively high education level of the participants in the validation sample, and that the instrument may be less sensitive to early cognitive impairment presented primarily in the form of nonamnestic deficits. Sensitivity and specificity were not determined, which limits the interpretation and utility of the test. It is indicated that longitudinal follow-ups are necessary to confirm the instrument's diagnostic value, to monitor rates of cognitive progression, and to identify which test variables best predict clinical conversion. The MATS can be also administered face-to-face by trained researchers/clinicians and it can be applied to individuals who cannot read and/or write and/or are visually impaired.

### Cognitive telephone screening instrument (COGTEL)

The COGTEL is a six components screening instrument that covers working, long-term verbal and prospective memory, verbal fluency and inductive reasoning (Kliegel et al., 2007). Its purpose is to assess cognitive-function domains across adulthood. The scores of the subtests can be analyzed one by one or be combined to a total score. Studies suggest that the COGTEL can be administered in large samples, including in cross-sectional, longitudinal and/or epidemiological (Kliegel et al., 2007), allowing for the global assessment of cognitive function among healthy younger and older adults test without being constrained by ceiling effects. The ongoing epidemiological study “Estrogen and Thromboembolism Risk (ESTHER)” represents an example of where it has been applied (Breitling et al., 2010). No information is provided concerning specific characteristics and/or qualifications of the applicants and/or interviewers.

### Telephone montreal cognitive assessment (T-MoCA)

In order to reduce missing data for patients that cannot be physically present in a clinical context, Pendlebury et al. (2013) developed a telephone version of the Montreal Cognitive Assessment (MoCA), a widely recognized brief screening test for milder forms of cognitive impairment (Nashreddine et al., 2005). The T-MoCA includes items that do not require the use of a pencil and paper or a visual stimulus, with the exception of the sustained attention task where subjects have to tap the side of the telephone with a pencil, instead of tapping on the desk. This tool, combined with the TICSM, was tested in a sample of community-dwelling patients 1 year after they had a transient ischemic attack (TIA) or a mild stroke. Overall, although abstraction, verbal fluency and repetition items may have been affected by telephone administration (patients performed worse), the T-MoCA proved to have sufficiently good accuracy to detect MCI among stroke/TIA patients (Pendlebury et al., 2013). The authors have also developed a shortened version of the T-MoCA (T-MoCA-Short) that includes only verbal fluency, verbal recall and orientation domains (Pendlebury et al., 2013). No information was provided regarding administration time or the T-MoCA(-Short) application in “healthy/normal” cognitive aging studies. Optimal cut-offs for the T-MoCA (and of the TICSM) will vary with different definitions of MCI.

## Concluding remarks

**Figure 2:**
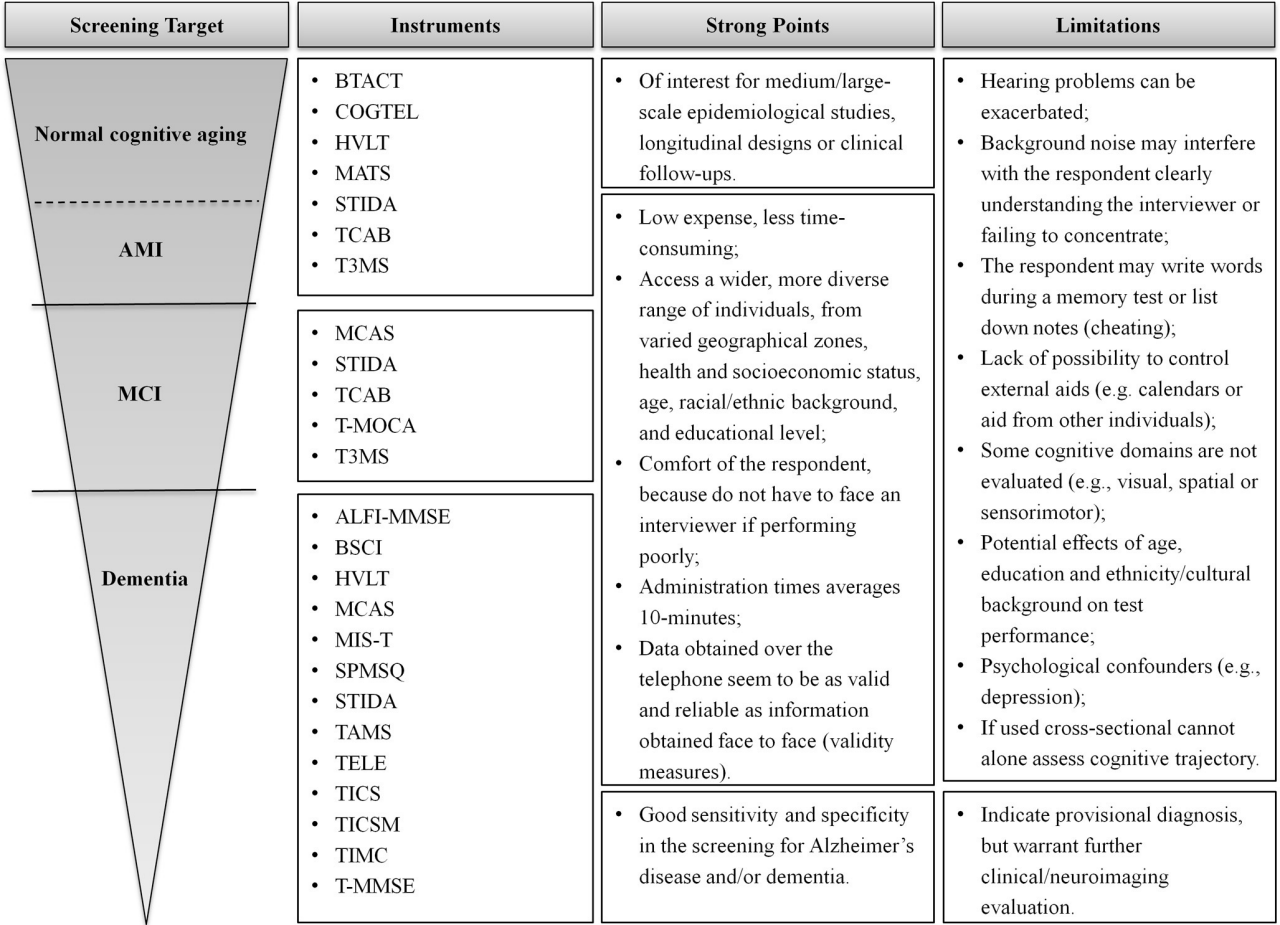
**Summary of the instruments for different screening goals**. Key advantages and limitations of each set of instruments are identified. The choice of an appropriate screening measure depends on the question being asked and the sample studied.

Screening for cognitive impairment is a relevant issue in clinical neuroscience and geriatrics. The use of cognitive assessments provides an important basis for diagnosing cognitive disorders and monitoring cognitive decline and, hence, disease progression. Specifically, the evaluation of cognition in healthy older/elderly individuals can help define the extent of alterations in cognition associated with normal aging, thus allowing a valid differentiation of “normal” (healthy cognitive aging) from “abnormal” (pathology-related cognitive decline/impairment) cognitive changes (Rapp et al., 2012). Here, culling from the available literature, we provide a compiled base of the available telephone-based instruments for neurocognitive screening, giving information on the instrument, its main characteristics, and validation measures (Table 1). Based only on published and peer-reviewed studies, a total of 19 validated instruments were identified, with 26.5% of which further validated in countries (and/or languages) other than the original. A summary of the instruments is shown in Figure 2, reporting on main advantages and limitations.

From the literature review, the TICS was identified as the most widely translated and validated instrument, which may be considered as a possible indicator of the extent of its applicability. Regarding the validation sample, most studies (31.6%) used a “mixed cohort” design; that is, instruments were applied in cohorts composed of both “healthy” individuals and those diagnosed with MCI, dementia, minor stroke, TIA, Alzheimer's disease (and/or Alzheimer's dementia, DAT) or dementia. These are altogether, hereafter, referred as “cognitively impaired.” A similar percentage of studies (31.5%) conducted either a random (21%, community-dwellers) and/or convenience (10.5%, geriatric outpatient programs) sampling, for which, in both cases, no previous cognitive performance diagnosis was known regarding if participants were “healthy” or “cognitively impaired”. The remaining studies focused either only on “healthy” (15.8%) or only on “cognitively impaired” (26.3%) individuals, with already previous indication for the cognitive status. From the total of *n* = 2315 patients/participants assessed, in the total of the 19 studies considered, a ratio of 2.62:3.04:4.34 was noted, respectively, “healthy”:“cognitively impaired”:“cognitively unknown” individuals. Largely, the instruments were applied in aging cohorts, with only the BTACT and the COGTEL having also been administered to younger cohorts.

Perhaps as one of the overall major shortcomings, several instruments lacked direct comparison with more extensive cognitive batteries and/or validation in other cohorts. In fact, regarding the former, only 15.8% of the studies used a combination of gold standards strategies for validation, with only one-fifth having conducted clinical assessment (or use of the CDR or the DSM-III-R) criteria to confirm assessment (“healthy” vs. “cognitive impairment”), and one-third using validated scales/instruments (other than the MMSE/3M) for the same purpose. It is particularly disturbing that 10% of the studies did not use (or reported) any type of gold standard for comparison or measure of validity, and a similar percentage did not describe the validation strategy utilized. The MMSE (or the 3M) was used as the gold standard in the majority of the studies (36.8%). This may warrant some considerations. Despite the fact that the MMSE is the most commonly used instrument for global cognitive screening (Molloy and Standish, 1997), possessing good reliability indexes, some of its limitations have been identified particularly in detecting subtle memory losses (Small, 2002). For instance, a subject with a low educational level may score poorly in the absence of cognitive impairment, while a subject with a higher educational level may score above threshold despite having cognitive impairment and/or respective decline in cognition (Brayne and Calloway, 1990; Tombaugh and McIntyre, 1992). As such, telephone-based cognitive screening tools have been mainly used to obtain a global cognition score and/or to address the lower end of the cognitive ability spectrum, but are unable to detect or indicate MCI onset and/or early phases of dementia, or indicate functional and psychological status. This is probably due to the fact that most instruments were at first developed to either detect dementia in normal samples (Crooks et al., 2005), and/or to facilitate Alzheimer's disease studies, rather than to identify at-risk individuals in diverse contexts (Crooks et al., 2005).

Feasibly, for validation purposes, and/or to guarantee the efficiency of the instrument in each subsequent study, a strategy to overcome the above mentioned shortcomings could be to conduct a “triage” screening assessment using a telephone-based tool and, in a parallel manner, accompany this with an in-person standard assessment using a comprehensive neurocognitive test battery. This battery should comprise domains from global cognition to information processing/attention, memory and executive function. This could be conducted in at least a percentage of the cohort, so to provide continued measures of internal validity. Some authors further suggest that telephone cognitive assessments could be preceded by an initial selection of subjects of interest made by postal questionnaires, providing further “triage” points depending on the research question of interest (Van Uffelen et al., 2007).

Interestingly, only for the MATS a longitudinal assessment was conducted in the validation study. This handicap should be addressed. The applicability of telephone assessment instruments should, after an initial assessment in a cross-sectional design, also be considered in a longitudinal approach if to better evaluate the sensitivity of the instrument for cognitive changes over time. A useful indication of the degree of cognitive decline can be gained by a combination of different methods in order to discriminate validly and reliably between healthy and “abnormal” mental aging across time (Mackinnon and Mulligan, 1998). For example, a combined approach of a telephone-based cognitive-screening instrument with a questionnaire based on informant reports [such as the Informant Questionnaire on Cognitive Decline in the Elderly, IQCODE (Jorm, 1994)] could be applied. Given their complementary characteristics, the combined use of telephone cognitive screening and informant reports, in cognitive evaluations, is expected to yield promising results in indicating for cognitive trajectories (Knafelc et al., 2003).

All studies considered reported on the statistical methodology used in the data analysis, with 68.4% reporting on both sensitivity and specificity measures. On average, the size of the cohorts was *n* = 121 individuals. Given that, on average, the number of items per instrument was 15, and considering that sample size should be 10 times the number of items for analysis (Nunnaly, 1978; Comery and Lee, 1992; Tabachnik and Fidell, 1992), overall the studies considered lacked by 15% in sample size for proper validation. When considered separately, approximately 50% of the studies had sample sizes that were too small for validation, with these on average needing to double participant number for full validation. Nonetheless, it should also be noted that while “10 subjects per item” is recommended when examining individual items, it is less clear if this applies when using the global score. Still, as some of the samples were small, making meaningful conclusions regarding some of the tests is difficult. A total of nine studies included cut-off scores indicative of “cognitive impairment.” This may overall be one of the most practical goals for the use of these instruments, placing them on similar ground with the rapid assessment tools already extensively used face-to-face. The average time of administration for the instruments considered was 10.8 min, with no administration time reported in six of the studies. Finally, common limitations were reported. The administration procedure could be particularly difficult for severely demented individuals, for those who had poor telephone communication skills or were hearing impaired and/or who were more easily distracted (shorter attention spans). Also, as tasks are instructed and solved verbally, those that involve visual, spatial, or sensorimotor skills cannot be evaluated. Finally, individuals may ask for the help of those nearby (or use external cues/aids) while performing the assessment (Smith et al., 2008). It is also overall recommended that future studies should evaluate for the test–retest and parallel-test reliabilities of the instruments.

As discussed, the current shift in population demographics has been accompanied by a need to develop brief and accurate cognitive screening instruments, with the potential to be applied in different research and clinical contexts for the cognitive assessment in medium to large-population samples (Petersen et al., 2001; Jager et al., 2003). As face-to-face administration tools require individuals to be physically present, telephone instruments have been developed as an alternative. If developed, used and validated properly, despite intrinsic limitations, telephone-based cognitive assessment can considerably contribute to increase sample sizes by reaching more individuals, and can also provide a mean for minimizing costs and participants' burden and accessibility. Furthermore, several studies demonstrated that the outcome in cognitive assessments administered by telephone is similar to that conducted in face-to-face settings (Wilson and Bennett, 2005). Furthermore, it is also noted that telephone-based tools can reduce selection bias in epidemiology studies by allowing covering large areas and facilitating follow-up (Herr and Ankri, 2013). A final comment concerns the use of telephone cognitive assessment for clinical diagnosis. Telephone-based cognitive instruments can be used as screening tools, but are inadequate tools for a diagnostic decision about the presence of MCI or dementia. Results warrant the same care in interpretation as if using face-to-face assessment with “rapid assessment” instruments (e.g., the MMSE). Telephone tools can be used for provisional early diagnosis, with later evaluation by accurate clinical examination, neuropsychological assessment, sources of information based on informant reports, and laboratory and imaging methods. In conclusion, telephone-based instruments should continue to be further developed and evaluated, and/or improved-on (namely in an era where distant video connection is emerging), in order to be utilized by health care professionals and researchers, serving as a viable complementary and/or alternative tool for cognitive screening in clinical and epidemiological settings.

### Conflict of interest statement

The authors declare that the research was conducted in the absence of any commercial or financial relationships that could be construed as a potential conflict of interest.
